# Practice characteristics and prescribing of cardiovascular drugs in areas with higher risk of CHD in Scotland: cross-sectional study

**DOI:** 10.1186/1475-9276-7-18

**Published:** 2008-07-15

**Authors:** Gary McLean

**Affiliations:** 1 Research Fellow, General Practice & Primary Care, Community Based Sciences, University of Glasgow, 1 Horselethill Road, G12 9LX, Glasgow, UK

## Abstract

**Background:**

We examine whether practices in areas with higher risks of CHD prescribe different levels of cardiovascular drugs and describe how they differ in GP and practice characteristics.

**Methods:**

Propensity score matching was used to identify two groups of practices in Scotland. The cases were in areas with 5% or more of the population in South Asian ethnic groups. The controls were in areas with less than 1% of the population in South Asian ethnic groups and were matched for other population characteristics.

**Results:**

The 39 case practices have lower prescribing rates than the matched controls for all heart disease drugs Significant different are found for six drugs (statins, ace Inhibitors, clopidogrel, thiazides, warfarin and digoxin. The differences range from 12.8% less for amlodipine to 43.9% for clopidogrel. The case practices also have lower prescribing costs than the unmatched group with the exception of ace inhibitors and aspirin. The highest prescribing costs for all drugs are found in the matched control group. The case practices are smaller than the controls, and have fewer GPs per 1,000 patients. Case practices have fewer quality markers and receive less in total resources, but have higher sums reimbursed to cover their employed staff costs.

**Conclusion:**

Patients with higher risk of CHD tend to live in areas served by practices with lower prescribing rates and poorer structural characteristics. The scale of the differences in prescribing suggests that health care system factors rather than individual treatment decisions cause inequity in care. Identifying whether South Asian *individuals *are less likely to receive heart disease drugs than non South Asians requires individual-level prescribing data, which is currently not available in the UK.

## Background

In 2003 CHD was second only to cancer as the major cause of mortality in Scotland. [1] Although CHD mortality has fallen in recent years death rates from CHD are amongst the highest in the world and the second highest in Western Europe. [2] There is a strong correlation between increasing incidence and mortality from CHD and deprivation. CHD is also the major cause of morbidity and mortality in the South Asian population in the United Kingdom. [3] South Asians have been found to be at increased risk compared to the rest of the population of England and Wales [4] by at least 40 percent. [5-7] Though Scotland has one of the worst incidences of heart disease in Europe [8] only one of the 19 studies identified in Bhopal's review was based in Scotland. [4,9]

The concept of equity is a central objective of most health care systems in the developed world. While governments from across the political spectrum, both in the UK and internationally, have attempted to tackle perceived inequities in health care the concept of equity remains somewhat elusive. [10,11] A theoretical framework has been set out which examines equity through three domains: equal access to health care for people in equal need; equal treatment for people in equal need; and equal outcomes for people in equal need. [11] This simple framework has been used as a basis to examine the equity of GP prescribing rates for statins and five major CHD drug groups focused around the equal treatment in equal need domain. [12,13] These papers are amongst a growing body of work in the UK, which have focused on equity of prescribing. However, these studies have largely been confined to England and Wales. The purpose of this paper is to explore the equity of prescribing for a range of heart disease drugs in Scotland. Having established prescribing differences, the analysis then considers structural differences in GP and practice provision. Using a matching technique, we use examine the notion of equal treatment for people in equal need and how this relates to differences in equal access to health care.

Many patients do not receive the appropriate treatment for CHD. Research has found that prescribing rates of statins and lipid lowering drugs were negatively correlated with deprivation. [9,14] The Acheson report highlighted the need for studies of ethnic inequalities. [15] Several studies have highlighted ethnic variations in access to and provision of hospital interventions. [16,17] Although a more recent study found no evidence that South Asian ethnicity was associated with lower use of cardiac procedures or drugs independent of clinical need, [18] there has been little research conducted on the equity of prescribing in the community.

One US study based on individual data discovered that black and minority ethnic group patients were less likely to be prescribed a beta-blocker. [19] There are no studies based on individual level data from the UK. Two studies in England have shown negative correlations between prescribing of lipid-lowering drugs [14] and beta-blockers [20] with the estimated proportion of patients from South Asian ethnic groups. Members of ethnic minorities tend to be situated in deprived areas and deprived areas have been shown to have lower quality and fewer general practitioner services than more affluent areas. [21-23] While a study in Scotland has found under the new GMS contract that achievement levels for the taking of beta blockers for patients with CHD, was found to be negatively associated with deprivation [24].

Since ethnicity data are not available on individual prescriptions, we compare prescribing rates for practices serving areas with higher proportions of South Asian patients to those serving areas with lower proportions of South Asian patients. Thus, we can use higher proportions of South Asian patients as a proxy for higher risk of CHD and then assess whether practices with populations associated with higher CHD prevalence have higher prescribing rates. We use a statistical matching process since practices differ in a range of other dimensions that may influence prescribing. Propensity score matching is a method for matching members of different groups based on a range of characteristics. Comparisons of the matched groups reveal the impact of the stratifying variable. The use of a matching process allows for the formation of groups based on their risk of having CHD, which can be assessed from their ethnicity, deprivation, social factors such as health and education and demographic factors. Comparisons are made in prescribing rates of a wide range of drugs used in the treatment of heart disease.

## Methods

National data are used to give 100% coverage of practices. Collection of characteristics of persons receiving prescriptions has only recently started to be piloted in the Scottish prescribing information system. It is therefore possible only to analyse variations in prescribing between practices (or higher organisational units). Ethnic compositions of practice populations are also not collected, so these must be estimated based on the ethnic compositions of the areas from which practices draw their populations.

The most recent and comprehensive information on the ethnicity of the Scottish population comes from the 2001 Census. Figures are produced for output areas (N = 42,604; average population = 117 persons) using the following ethnic groups: White (97.9%); Indian (0.3%); Pakistani and Other South Asian (0.9%); Chinese (0.3%); Other (0.6%).

We combined the Indian and Pakistani and Other South Asian groups and calculated the proportion of each output area's population from South Asian ethnic groups. We calculated the proportion of each practice's list resident in each output area as at September 2002 using an extract from the Community Health Index. We attributed the South Asian proportions to practices using this geographical breakdown to estimate the likely South Asian proportion of each practice's list. This process assumes that practices draw representative samples of individuals from the output areas.

We analysed the characteristics of patients and practices that were associated with the estimated South Asian proportion. We used a binomial logit multiple regression model [25] to identify the significant population characteristic predictors used to match the GP practices. [26-28] As well as providing an epidemiological analysis of the geographical distribution of South Asians in Scotland, this allowed us to generate a *propensity score *for each practice, representing the expected South Asian proportion given the other population factors with which it is significantly correlated. [29]

We compared the prescribing rates for the cases (practices with South Asian proportions over 5%) with figures for matched controls and unmatched controls. These figures are weighted averages where the weights represent the propensity of each control to match the cases. The propensity score matching results were estimated using STATA v8.2. We used the Kernel matching method [30], though the results are similar with other options. We estimated standard errors via bootstrapping with 100 replications.

We analysed data for a wide range of drugs used in the treatment of heart disease, including: statins, beta blockers, aspirin, warfarin, ACE inhibitors, clopidogrel, thiazide, digoxin, spironolactone and amlodipine. Prescribing rates were measured by age and sex standardisation of total Gross Ingredient Costs in 2001/2 by Specific Therapeutic group age-sex weightings related prescribing units (Star_PUs) for cardiovascular drugs, with the exception of lipid-lowering drugs for which we had Defined Daily Doses.

We also obtained a set of GP and practice characteristics from the General Medical Practitioner Database for October 2002 and GMS payments made to practices in the 2002/3 financial year. The Royal College of General Practitioners (RCGP) supplied lists of practices that had received Practice Accreditation (PA) or the Quality Practice Award (QPA) by the end of 2002.

Five variables were used in the matching equation covering deprivation, health rurality and number of temporary residents. Deprivation was measured by the Carstairs score, derived at output level. The Carstairs score is an unweighted sum of *z*-scores for four variables representing car ownership, male unemployment, social class and overcrowding. For health, two indirectly standardised variables taken from the Census representing the age sex standardised ratio for limiting long term illness and not good health were used. Variables representing the proportion of temporary residents and number of patients qualifying for a road mileage payment were also used.

## Results

Table 1 lists the variables used for matching and the matching equation. The table shows that the matching equation indicates that the cases group is significantly different from practices with less than 1% of the population with South Asian patients for all the variables with the exception of the number of temporary residents. Table 1 also shows that the matching process leaves us with a matched control group of 140 practices. The matched control group has higher deprivation and morbidity scores but the differences are not statistically significant. Both the cases and matched control group have higher deprivation and morbidity scores than the unmatched control group.

**Table 1:** Population characteristics and the matching equation

Variable	Mean Values		Coefficient	P-value	Mean Value Matched Controls (SA<1%)	Difference Between Cases And Matched Control P value	Mean Value Unmatched Controls (SA<1%)
						
	All	Cases (SA>5%)					
Number of Practices	702	39			140		523
Proportion of South Asian patients	0.01	0.10	-	-	0	<0.001	
Carstairs	-0.17	2.3	0.45	<0.001	2.6	0.22	-0.7
Census not good health	98.9	122	0.11	<0.001	134	0.12	91.4
Census limiting long term illness	98.8	107	-0.2	<0.001	121	0.09	95.2
Temporary residents/practice list size	0.03	0.01	-3.23	0.22	0	0.31	0.05
Proportion of list eligible for road mileage payments	0.11	0	-2.88	<0.001	0.01	0.15	0.19

Notes: Analysis weighted by list size. Dependent variable in the matching equation is the log-odds of the population proportion in South Asian ethnic groups. Adjusted R-squared for the model = 0.59* Figures for matched controls obtained by weighted average using kernel matching.

**Table 2:** Prescribing rates for CHD drugs

Drug	Star-PUs				
	Cases	Controls		Difference [95% C.I.]	% Difference

		Unmatched	Matched		

Statins* (BNF Chapter 2, 2.12)	98.9	99.4	136.7	-37.8 [-66.7 to -11.5]	-27.7%
Ace Inhibitors (BNF Chapter 2, 2.5.5)	104.7	97.3	144.4	-39.7 [-64.3 to -14.5]	-27.5%
Amlodipine BNF Chapter 2, 2.6)	89.2	107.0	102.2	-13.0 [-44.3 to 13.4]	-12.8%
Beta blockers (BNF Chapter 2, 2.4)	92.7	101.9	106.2	-13.5 [-38.9 to 9.9]	-12.7%
Clopidogrel (BNF Chapter 2, 2.9)	86.6	99.6	154.3	-67.6 [-105.8 to -17.8]	-43.9%
Thiazide ((BNF Chapter 2, 2.5)	92.7	103.4	134.8	-42.2 [-62.7 to -23.6]	-31.3%
Aspirin (BNF Chapter 2, 2.9)	109.0	102.2	136.2	-27.2 [-57.7 to 3.6]	-20%
Warfarin (BNF Chapter 2, 2.8)	77.2	100.2	101.7	-24.5 [-38.1 to -6.5]	-24.1%
Spironolactone (BNF Chapter 2, 2.5)	87.0	100.3	109.8	-22.9 [-63.9 to 0.6]	-20.8%
Digoxin (BNF Chapter 2, 2.1)	92.0	100.9	122.6	-30.6 [-55.7 to -9.6]	-25%

Notes: * measured in Defined Daily Doses

For all CHD drugs, case practices have lower prescribing costs than the matched controls. Significant different are found for six drugs (statins, ace Inhibitors, clopidogrel, thiazides, warfarin and digoxin. The differences range from 12.8% less for amlodipine to 43.9% for clopidogrel. The case practices also have lower prescribing costs than the unmatched group with the exception of ace inhibitors and aspirin. The highest prescribing costs for all drugs are found in the matched control group. However, this does not account for differences in deprivation and morbidity between the matched and unmatched controls group.

Table 3 shows that the cases group has nearly double the proportion of GPs over 55 than the matched control. Practices in the case group have significantly fewer GPs per practice (2.9 to 3.9) and receive less through performing minor surgery. There are no QPA or PMS practices in the cases group. The cases group have significantly fewer GPs (0.57 to 0.66) and WTE GPs (0.58 to 0.73) per 1000 of the population than the matched controls group. The cases group also receive significantly lower GMS payments (47.9 to 53.4)

**Table 3:** Practice and GP characteristics

Variable	Cases	Controls		Difference [95% Confidence Interval]	Difference %
				
		Unmatched	Matched		
*GP characteristics*					

Proportion of female GPs	0.37	0.34	0.35	0.02 [-0.06 to 0.12]	5.7%
Average age of GPs	46.2	45.5	44.1	2.1 [-1.6 to 3.5]	4.7%
Proportion of GPs over 55 years	0.19	0.12	0.10	0.09 [0.03 to 0.16]	90%

*Practice size*					

Numbers of GPs per practice	2.9	4.9	3.9	-1.0 [-1.7 to -0.4]	-25.7%
Single-handed practice	0.23	0.11	0.11	0.12 [-0.07 to 0.21]	109%
One or two partner practice	0.44	0.35	0.27	0.17 [-0.07 to 0.31]	62.9%

*Services offered*					

Practice does not offer minor surgery	0.18	0.04	0.08	0.10 [-0.04 to 0.19]	125%
Minor surgery payments (£ per capita)	0.67	0.92	0.82	-0.25 [-0.42 to -0.09]	-18.3%
Night visit claims per capita	0.03	0.03	0.03	-0.00 [-0.002,0.004]	0.0%

*Quality markers*					

Training practice	0.18	0.25	0.21	-0.03 [-0.2, to 0.01]	-14.3%
Practice Accreditation	0.19	0.25	0.24	-0.05 [-0.16 to 0.14]	-20.9%
Quality Practice Award	0.00	0.06	0.04	-0.04 [-0.06 to -0.01]	100%
Personal Medical Services	0.00	0.03	0.06	-0.06 [-0.10 to -0.02]	100%

*Resources/workload*					

GPs per 1000 population	0.57	0.88	0.66	-0.09 [-0.14 to,-0.03]	-15.7%
WTE GPs per 1000 population	0.58	0.90	0.73	-0.15 [-0.29 to -0.02]	-21.6%
Total GMS payments	47.9	57.8	53.4	-4.5 [-6.1 to -2.2]	-10.3

The unmatched control group has the highest number of GPs per practice and higher number of GP and WTE per 100 of the population. They are more likely to be a training, PA or QPA practice. Practices in the unmatched control group also receive more through minor surgery and GMS payments than the other two groups.

## Discussion

This paper has examined equity of prescribing for a range of heart disease drugs in Scotland. We have found notable differences between practices serving areas with more than 5% of the population in South Asian ethnic groups and those with similar characteristics, but serving populations with less than 1%.

Previous research has consistently found that patients from South Asian ethnic groups have higher levels of CHD and consequently a greater need for provision and quality of health care. Thus, the findings of this study would suggest that prescribing rates and provision of care in Scotland are inequitable and that the inverse care law still applies. [10,23]

### Possible explanations and implications

Scotland has higher overall rates of CHD than the rest of the UK and most European countries as evidenced by available mortality and morbidity statistics. [2,31] South Asian populations in the UK have a younger age profile than average leading to lower CHD rates and lower expected rates of prescribing. [32,33] However, our case practices are concentrated in one Scottish NHS Board and this Board has the highest recorded rates of CHD in Scotland. [34,35] Moreover the cases group has higher levels of deprivation than the unmatched control group. Thus prescribing should be higher in the cases group if deprivation is related to CHD prevalence. But we found higher prescribing for all drug groups in the unmatched control group for all but two of the indicators.

The degree to which differences in practice characteristics can explain our prescribing results is mixed. Case practices have fewer numbers of GPs who tend to work longer hours than those in the matched control group. This is of concern since South Asian patients have higher than average consultation rates. [36] Consequently a greater workload is placed on a fewer number of doctors within the cases group and lower prescribing rates have been found to be positively associated with lower ratios of GPs to patients. [37] Moreover, research into aspects of quality of care have emphasised the importance of adequate time for consultations with the view that GPs require greater time to be allowed to treat complex diseases such as CHD in the proper manner. [36,38] There is little evidence with regard to the impact on prescribing rates for practices with accreditation. However, there is some evidence that training practices, of which there are far fewer in the cases group, have lower prescribing rates than non-training practices. [39,40]

### Strengths and weaknesses of the study

This study has the advantage of 100% coverage since the data are available for all practices. At the time the study was conducted other than for statins only cost data was available to the authors. Previous research has suggested that using prescribing rates based on prevalence rates is more beneficial than derived from costs patterns. [41] However volume remains the main driver of prescribing costs and needs based formulas adopted in England for prescribing expenditures have been based on net ingredient cost. [38]. This study has the advantage of 100% coverage since the data are available for all practices. The findings are consistent across a wide range of drugs used in the treatment of heart disease. Lower levels of prescribing are observed relative to all other practices and to a set of matched controls. However, the analysis is ecological and the magnitude of the differences suggests that the results cannot be explained wholly by relative under-prescribing to South Asian *individuals *but rather is indicative of prescribing of practices in more deprived areas. We have estimated more than 20% differences in prescribing rates, yet South Asians represent only 9% of the population in the cases group. Taking account of the higher prevalence rate in South Asians suggests that no more than 11% of heart disease patients are South Asian. Even if *no *South Asian patients receive treatment and *all *non-South Asian patients receive treatment, this cannot account for the 20% difference in prescribing. Our results must therefore reflect, at least in part, *contextual *influences rather than *compositional *characteristics alone. [37] Our comparison of structural factors suggests that there are considerable differences in the structure of care delivery that may constitute part of these contextual influences.

## Conclusion

This study shows that South Asians tend to be registered with practices with lower prescribing rates, but it would appear that all patients in these practices are at greater risk of having lower prescribing rates. Moreover, patients in these practices also suffer from poorer access to and lower quality GP services. Understanding the structural, attitudinal or behavioural reasons for lower prescribing in these practices and how these are affected by GP and practice provision is a challenge for future work. Identifying whether South Asian *individuals *are less likely to receive heart disease drugs than non South Asians will require individual-level prescribing analysis. Our results suggest that it will be important to identify the role of practice-related factors.

## Competing interests

The author declares that he has no competing interests.
